# Supplementary material for: AffyMAPSDetector: a software tool to characterize Affymetrix GeneChip™ expression arrays with respect to SNPs
Source: BMC Bioinformatics. 2007 Jul 30;8:276. doi: 10.1186/1471-2105-8-276 (PMC1959249; doi:10.1186/1471-2105-8-276)
Supplement: Additional file 13 — AffyMAPSDetector v1 source code. AffyMAPSDetector source code. [file 1471-2105-8-276-S13.zip › AffyMAPSDetector_v1_src/doc/affychip/snpdetector/util/PmMmHeatmapViewerModel.html]

PmMmHeatmapViewerModel


|  |  |  |  |
| --- | --- | --- | --- |
| **Overview** \| **Package** \| **Class** \| **Use** \| **Tree** \| **Deprecated** \| **Index** \| **Help** | | |  |
| **PREV CLASS**   **NEXT CLASS** | **FRAMES**    **NO FRAMES**     **All Classes** |  |  |
| SUMMARY: NESTED \| FIELD \| CONSTR \| METHOD | DETAIL: FIELD \| CONSTR \| METHOD |  |  |


---


## affychip.snpdetector.util Class PmMmHeatmapViewerModel

```
java.lang.Object
  affychip.snpdetector.util.PmMmHeatmapViewerModel
```

---

public class **PmMmHeatmapViewerModel** extends java.lang.Object

Title: Probe SNP Detector

Description: This class serves the purpose of data model underlying the
HeatmapViewer GUI. The table(s) and the list(s) in the heatmap view should
use an instance of this class as their respective data model.

Copyright: Sunita Kumari -- Copyright (c) 2004

Company: George Mason University

**Version:**
:   1.1

**Author:**
:   Sunita Kumari

---

|  |  |
| --- | --- |
| **Nested Class Summary** | |
| `class` | `PmMmHeatmapViewerModel.Row`             Title: Probe SNP Detector |
| `static class` | `PmMmHeatmapViewerModel.RowComparatorForDataSorting` |


|  |  |
| --- | --- |
| **Field Summary** | |
| `protected  java.util.Vector` | `columns` |
| `protected  java.util.Vector` | `dataModel` |
| `protected  double` | `maxMm` |
| `protected  double` | `maxPm` |


**Constructor Summary**

`PmMmHeatmapViewerModel()`

`PmMmHeatmapViewerModel(java.io.File pmMmMatrixFile)`

`PmMmHeatmapViewerModel(java.lang.String pmMmMatrixFilename)`


|  |  |
| --- | --- |
| **Method Summary** | |
| `void` | `setPmMmMatrixFile(java.io.File pmMmMatrixFile)`             This method loads the PM/MM ratio matrix file and builds the data model for the heatmap viewer. |
| `void` | `setPmMmMatrixFile(java.lang.String pmMmMatrixFilename)` |

**Methods inherited from class java.lang.Object**

`clone, equals, finalize, getClass, hashCode, notify, notifyAll, toString, wait, wait, wait`

**Field Detail**

### columns

```
protected java.util.Vector columns
```

---


### dataModel

```
protected java.util.Vector dataModel
```

---


### maxPm

```
protected double maxPm
```

---


### maxMm

```
protected double maxMm
```


**Constructor Detail**

### PmMmHeatmapViewerModel

```
public PmMmHeatmapViewerModel()
```

---


### PmMmHeatmapViewerModel

```
public PmMmHeatmapViewerModel(java.lang.String pmMmMatrixFilename)
                       throws java.lang.Exception
```

---


### PmMmHeatmapViewerModel

```
public PmMmHeatmapViewerModel(java.io.File pmMmMatrixFile)
                       throws java.lang.Exception
```


**Method Detail**

### setPmMmMatrixFile

```
public void setPmMmMatrixFile(java.lang.String pmMmMatrixFilename)
                       throws java.lang.Exception
```

:   **Throws:**: `java.lang.Exception`

---


### setPmMmMatrixFile

```
public void setPmMmMatrixFile(java.io.File pmMmMatrixFile)
                       throws java.lang.Exception
```

:   This method loads the PM/MM ratio matrix file and builds the data model
    for the heatmap viewer.

    :   **Parameters:**: `pmMmMatrixFile` - type java.io.File - The PM/MM ratio matrix file. **Throws:**: `java.lang.Exception`


---


|  |  |  |  |
| --- | --- | --- | --- |
| **Overview** \| **Package** \| **Class** \| **Use** \| **Tree** \| **Deprecated** \| **Index** \| **Help** | | |  |
| **PREV CLASS**   **NEXT CLASS** | **FRAMES**    **NO FRAMES**     **All Classes** |  |  |
| SUMMARY: NESTED \| FIELD \| CONSTR \| METHOD | DETAIL: FIELD \| CONSTR \| METHOD |  |  |


---
